# Surgery and universal health coverage: Designing an essential package for surgical care expansion and scale-up

**DOI:** 10.7189/jogh.10.020349

**Published:** 2020-12

**Authors:** Ché L Reddy, Dominique Vervoort, John G Meara, Rifat Atun

**Affiliations:** 1 Program in Global Surgery and Social Change, Harvard Medical School, Boston, Massachusetts, USA; 2 Department of Global Health and Social Medicine, Harvard Medical School, Boston, Massachusetts, USA; 3 Department of Plastic and Oral Surgery, Boston Children’s Hospital, Boston, Massachusetts, USA; 4 Johns Hopkins Bloomberg School of Public Health, Baltimore, Maryland, USA; 5 Department of Global Health and Population, Harvard T.H. Chan School of Public Health, Boston, Massachusetts, USA

Five billion people worldwide lack access to safe, timely, and affordable surgical and anaesthesia care, resulting in over 18 million deaths each year and one-third of the global burden of disease. In 2015, the World Health Organization and the Member States recognised surgical and anaesthesia care as a component of universal health coverage (UHC). National Surgical, Obstetric, and Anaesthesia Plans (NSOAPs) are long-term, strategic plans being developed by several low- and middle-income countries to strengthen emergency and essential surgical services by embedding within the government’s broader plans to implement UHC. Crucial, however, is the need for countries to define which surgical services should be included in essential health service packages. An approach that prioritises populations with the greatest need is vital for achieving financial risk protection and equity in global health. NSOAPs uniquely cross-cut health systems, allowing for the convergence of emergency and essential surgical care with other essential health services to meet broader UHC objectives.

How should surgical care (including obstetrics and gynaecology, anaesthesia, and the whole surgical ecosystem) be integrated within universal health coverage (UHC)? What surgical procedures should be included in essential health care packages? Who will receive such services? Who will pay? These are some of the questions that confront governments seeking to improve surgical care through UHC. These questions are not only daunting from a health systems perspective but also because of the scale of governmental challenges in increasingly uncertain political, economic, and socio-cultural contexts. The third Sustainable Development Goal (SDG Target 3.8) identifies UHC as a target for countries to attain by 2030 [1]. Surgical care is an integral component of UHC [2]: one-third of the global burden of disease, it is estimated, requires surgical intervention; 18 million people die from surgically treatable conditions annually [3]; and an additional 4.2 million die within 30 days of a surgical procedure each year [4,5]. There is an imperative to provide *access to safe, timely, and affordable surgical services* as part of UHC to achieve equity in global health systems.

## IMPROVING THE AVAILABILITY OF SAFE, TIMELY, AFFORDABLE, AND EQUITABLE SURGICAL SERVICES AS PART OF UNIVERSAL HEALTH COVERAGE

Although the disparities in access to surgical care around the world were described in 1980 as the “*most serious manifestation of social inequity in health care*” by Dr Halfdan Mahler, former Director-General of the World Health Organization (WHO) [6], it was not until 2015, when the Lancet Commission on Global Surgery released its report *Global Surgery 2030,* that the magnitude of these disparities was quantified [3]. The Commission estimated that five billion people lacked access to safe, timely, and affordable surgical and anaesthesia care when needed [1]. In 2015, the WHO Member States adopted the World Health Assembly (WHA) Resolution WHA68.15 “*Strengthening emergency and essential surgical care and anesthesia as a component of universal health coverage*” to improve surgical care worldwide [7].

**Figure 1:**
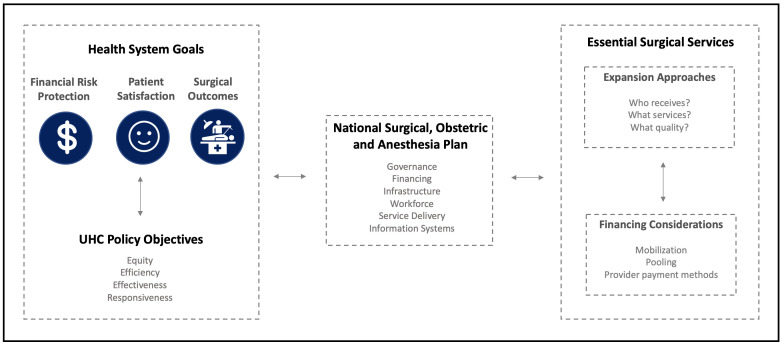
Framework: integrating surgical care within universal health coverage.

The objectives of providing robust surgical care incorporated within UHC are three-fold (**Figure 1**): First, to increase the level and distribution of health through the provision of safe and timely surgical care to all that need it. Second, to protect individuals from financial risk and impoverishment as a result of paying for surgical services. Third, to ensure that the quality surgical services provided are commensurate with what citizens need. At present, few countries meet all three surgical system objectives. Even the USA, a high-income country that spends 17% of its Gross Domestic Product (GDP) [8] on health, has yet to achieve these three objectives. UHC presents an opportunity to achieve these objectives.

## MOBILISING POLITICAL SUPPORT FOR INTEGRATING SURGICAL CARE WITHIN UNIVERSAL HEALTH COVERAGE

Effective UHC requires the provision of a range of quality health care services, of which surgical care is an essential element. Four questions need consideration when designing an essential package of surgical services and its financing as part of UHC:

1. *What* surgical services should an essential health service package for UHC include?
2. *Who* will receive the essential surgical package first, and which population groups will benefit thereafter?
3. *How* will the services be expanded and scaled-up to ensure universal coverage of surgical services*?*
4. *Who* will pay for these services and *how*?

Many countries implementing UHC do not adequately consider surgical services as an integral component of an essential health package. Benefits packages tend to focus on specific interventions, for example Cesarean section, rather than surgical systems as a whole, which means that even specific interventions are often not effectively implemented. Evidence demonstrating the critical need for surgical care, the feasibility of scaling-up surgical services, and the potential health and economic benefits need to be combined with political support to ensure inclusion of surgical care in UHC policy and implementation. Garnering political support requires understanding the political dynamics within countries, the reasons why specific health policy goals are pursued, and how these internal factors are influenced by changes in the broader global political economy. In turn, development of such an understanding requires an analysis of the stakeholders involved in shaping policies which are included in domestic political agendas and the process through which decisions are reached. These stakeholders are myriad but typically include political players, such as politicians and political parties, civil society, the electorate at large, professional associations, academic institutions, providers of health care services, the private sector, international organisations such as the WHO and the World Bank, and, among others, international development partners, which provide overseas development assistance for health.

## NSOAPS: A LINK BETWEEN SURGICAL SYSTEM NEEDS AND DEFINING THE SURGICAL PACKAGE

Many LMICs have developed systematic plans to include surgery within national health priorities and UHC [9-11]. National Surgical, Obstetric, and Anesthesia Plans (NSOAPs) outline a roadmap detailing how surgical care could be improved [7]. In addition to countries, regional organisations have also been involved in the development and adoption of NSOAPs. For example, recently, the Southern African Development Community (SADC), comprising 16 Member States in Africa, ratified an intergovernmental resolution to improve surgical care in the region and emphasised the need for countries to develop NSOAPs [12].

A principal objective of the NSOAP is to provide direction on each of the four surgery-UHC related policy objectives noted above through a systematic analysis of a country’s surgical system. The NSOAP process is important because it enables the knowledge base, technical expertise, and political capital needed to include an essential surgical package within UHC. Typically, the ministries of health oversee the development process for NSOAPs. The process emphasises inclusion and accountability, with the involvement of all relevant stakeholders who will be influenced by the policy [13-15]. An inclusive and transparent process helps to garner political support, develop legitimacy, reduce policy resistance and encourage acceptance of the health system reform effort during the implementation of the NSOAP. The process also involves the contribution of technical experts in various disciplines, including public health, economics and health policy, together with clinicians and epidemiologists, to generate the evidence and knowledge required to address the health system gaps in the workforce, infrastructure, information systems, service delivery, information management and governance that collectively influence the delivery of surgical care [15]. Established global surgery research networks could help ensure that NSOAP policies are grounded in evidence that reflects contextual specificities. GlobalSurg, for instance, is a collaborative research network funded by the National Institute of Health Research. The network involves more than 5000 clinicians across over 100 countries in research to evaluate surgical outcomes in LMICs [16]. The NSOAP process could leverage such networks and harness relevant research to enhance NSOAP formulation, implementation and impact evaluation. While NSOAPs provide the evidence for making rational investment cases for surgery and the technical approaches that could be pursued to scale-up services, they are less developed in providing the political pathways and strategies for persuading decision-makers to include essential surgical packages in UHC.

## Developing an essential surgical package for Universal Health Coverage

What strategies could countries adopt to include surgery in UHC? There are two strategic considerations concerning this question. The first relates to the mode of expansion of high-quality surgical services and the second to the fiscal space needed to fund this expansion.

The first strategic consideration, expansion of high-quality surgical services, could be explored using three expansion pathways: (i) to expand coverage before expanding the scope of surgical services – an approach that entails providing essential high-quality surgical services (eg, the Bellwether procedures – acute, high-value procedures and because their consistent provision is suggestive of functional surgical systems with broad service delivery [3] – such as open fracture repair, laparotomy, and cesarean delivery or select cost-effective high-impact procedures that have been recommended by the Disease Control Priorities 3) to all citizens, before expanding the scope to include other procedures (eg, cataract surgery, hernia repairs and so on); (ii) to expand the scope of services before expanding coverage of high-quality surgical services – eg, providing procedures in addition to the three Bellwether procedures to some population groups (eg, those insured or covered through a government-financed scheme), before making them available to other population groups, such as the uninsured, and (iii) to expand coverage of high-quality surgical services and scope simultaneously. Quality is of paramount importance when expanding the scale and scope of surgical services. An NSOAP explores the financial, technical, and political feasibility and the pros and cons of each of these expansion pathways for a particular context.

**Figure:**
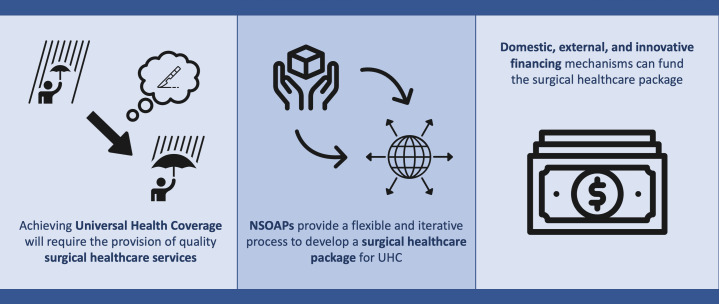
Photo: From the author’s own collection, used with permission.

The second strategic consideration relates to financing the expansion of surgical services. The financing decision revolves around mobilising additional funds, pooling mechanisms, and payment configurations.

Expansion of surgical services requires an increase in inputs (human resources, supplies, infrastructure, and knowledge) and outputs (additional surgical procedures provided with quality), which need to be funded by expanding fiscal space available for the health sector. Fiscal space refers to the capacity of a country to fund government expenditures, including for health [17,18]. Expansion of fiscal space (**Table 1**) is influenced by; (i) macroeconomic conditions; (ii) government budget allocation to the health sector; (iii) allocation of health sector-specific resources to priorities and interventions; (iv) improved health system efficiency that will lead to savings in the budget (for example more efficient procurement or supply chain management); (v) funding from external sources [19], and; (vi) innovative financing [20], such as international solidarity levy airline [20]. By evaluating and optimising each area that could create fiscal space, policymakers can determine an overall financial strategy to mobilise additional funding for expanding the essential surgical package.

**Table 1:** Components of fiscal space

Component	Example
Macroeconomic conditions	Sustained economic growth, allows increased budget allocation towards health
Reprioritization of government budget	Shifting the national budget allocation, allows for an increased health budget
Health sector specific resources	Instituting specific governmental revenue streams (eg, taxes) to increase health spending
Efficiency of existing resources	Improving the efficiency of health sector spending
External sources	Funding originating from international bilateral, multilateral, and philanthropic entities
Innovative financing	Novel financial mechanisms that mobilize, pool, and allocate funds to the health sector to produce greater value and impact. Eg, Global Fund

Pooling refers to how funds are collected and consolidated to provide solidarity and financial protection by reducing the risk of unpredictable catastrophic and impoverishing health care expenditures for individuals. Each year, 81.2 million people are impoverished seeking surgery, due to medical (ie, procedural and hospitalisation) and non-medical costs (ie, food, transportation, and opportunity cost)[3], which impacts on livelihoods of families and their ability to participate in economic and social life. The pooling of financial resources from multiple sources (eg, taxes, innovative financing, and other government revenues) into a single funding source increases solidarity by cross-subsidising risk across age, socio-economic position and disease groups.

Different payment methods can be used to remunerate health care providers, influence their performance at work, and ultimately, the quality of outputs and health services provided through the health system. Commonly used payment methods include (i) Fee-for-service, where providers are remunerated for each health service they provide; (ii) Performance-based or pay-for-quality method, whereby providers receive additional payments for improved health outcomes; (iii) Payment for bundled groups of services linked to diagnosis (eg, Diagnosis-Related Groups), and; (iv) Capitation payments, where providers are remunerated based on the number of enrollees for a given period, irrespective of the volume of health services used in that period. These methods can be used separately, or in combination, to support an expansion strategy for surgical services. For example, if the objective is to improve the quality of surgical services in a context where surgical volume is sufficient, a performance-based method could be used to create incentives for improved performance. Where service volume is low, a fee-for-service method may be appropriate to increase the supply of surgical services, while ensuring that the quality of services is not compromised. The selection of provider payment method will be influenced by both the desired change and the feasibility of implementing a particular method. For instance, performance-based payment methods will require more complex data and governance systems to collect, analyse, monitor and evaluate the metrics selected for performance, and capability to estimate the level of remuneration for performance achieved.

## INTEGRATING SURGICAL CARE WITHIN UNIVERSAL HEALTH COVERAGE – A CONCEPTUAL FRAMEWORK

**Figure 1** shows how the health system goals, UHC objectives, NSOAP process, and essential surgical service package relate and interact. The NSOAP, which is a strategic process, provides the link between health system goals, UHC objectives and the essential health service package that needs incorporating in UHC. An essential surgical package is developed through the NSOAP process, which helps attain UHC objectives and health system goals. UHC itself also enhances the attainment of health system goals. Including an essential surgical package in UHC must take into account both the expansion approach and the financing of the expansion approach considered. All three elements – health system, UHC, and the surgical package – interact in a context of dynamic complexity, and continually influence the health system and the delivery of surgical services.

The inclusion of an essential surgical package in UHC will require a systematic assessment of the health system goals and context, UHC objectives, and the country policies to achieve them, as well as an examination of the key considerations necessary to incorporate an essential surgical package in UHC – namely, what is included in the essential surgical package, and how it will be expanded and financed. The NSOAP can enable consideration of each of these dimensions while exploring the technical, economic and political feasibility of different approaches for incorporating essential surgical services within UHC. This approach also ensures that surgical services are not developed in isolation but are fully embedded within UHC.

For most LMICs, achieving UHC will require the inclusion of an essential high-quality surgical package. The NSOAP approach provides a strategic process for Ministries of Health to determine how to improve surgical care as part of UHC, within their established institutional and governance arrangements, taking into account health system capabilities.
